# Impact of Metal Nanoparticles on the Phytochemical and Antioxidative Properties of Rapeseed Oil

**DOI:** 10.3390/ma16020694

**Published:** 2023-01-10

**Authors:** Magdalena Kachel, Małgorzata Stryjecka, Lidia Ślusarczyk, Arkadiusz Matwijczuk, Iwona Budziak-Wieczorek, Grzegorz Gładyszewski

**Affiliations:** 1 Department of Machinery Exploitation and Management of Production Processes, Faculty of Production Engineering, University of Life Sciences in Lublin, 20-612 Lublin, Poland; 2 Institute of Human Nutrition and Agriculture, State Academy of Applied Sciences in Chełm, 22-100 Chełm, Poland; 3 Department of Biophysics, University of Life Sciences in Lublin, Akademicka 13, 20-950 Lublin, Poland; 4 Ecotech Complex Analytical and Programme Centre for Advanced Environmentally-Friendly Technologies, Maria Curie-Sklodowska University, 20-612 Lublin, Poland; 5 Department of Chemistry, University of Life Sciences in Lublin, Akademicka 15, 20-950 Lublin, Poland; 6 Department of Applied Physics, Lublin University of Technology, Nadbystrzycka 36, 20-618 Lublin, Poland

**Keywords:** antioxidants, ATR-FTIR spectroscopy, copper, fatty acids, nanoparticles, phytochemicals, silver

## Abstract

The agricultural uses of nanoparticles continue to be considered as innovative methods that require more in-depth research into their impact on product quality. In our study, we investigated the effects of fertilizers containing metal nanoparticles (silver AgNPs and copper CuNPs) during the plant growth stage of winter rape cultivation, and in most experimental variants, with the exception of the (x2) application of AgNPs, we observed a decrease in the mass of one thousand seeds (MTS). The obtained result was 11.55% higher relative to the control sample in 2019, and also increased after the (x1) 4.36% and (x2) 11.11% application of CuNPS in 2020. The analyzed oxidative stability of the oil increased in both experimental years (2019–2020), with the highest values recorded after the (x1) and (x2) application of CuNPS—4.94% and 8.31%, respectively, in the first year of cultivation, and after the (x2) application of CuNPS—12.07% in the subsequent year. It was also observed that the content of polyphenols, flavonoids, squalene, tocopherols α and δ, chlorophylls, and carotenoids increased in the oil. Moreover, spectral FTIR analysis was performed on the oil samples obtained from cultivations sprayed with solutions containing Ag or Cu nanoparticles and revealed changes in several spectral regions with the maxima at ~1740, 1370, 1230, and ~1090 cm^−1^. Additionally, a FTIR analysis conducted in combination with multivariate analysis allowed us to classify the studied oils into the most similar groups and to study the structure of data variability. The conducted analyses revealed that the use of nanoparticles resulted in decreased size of the produced seeds and improved antioxidative properties of rapeseed oil.

## 1. Introduction

The use of a variety of plant support technologies during crop growth can often significantly improve the quality of the end product. The available solutions include a selection of traditional and alternative fertilizers. In this context, nanotechnology is becoming increasingly common in agriculture with nanoparticles being commonly used in fertilizers, pesticides, bactericides, and fungicides. News [1] reports based on projections suggest that the nontechnological industry will grow by USD 124.7 billion, at a rate of 22.6%, by 2027. Newly emerging solutions will facilitate more extensive uses of noncompounds in, for example, pharmaceutic, cosmetic, profitable energy storage systems or agricultural production [2,3]. The nanoparticles (NPs) most commonly used in such contexts include a range of inorganic compounds: CuNP, AlNP, AgNP, zinc oxide (ZnO), Silica NP (SiNP), cerium oxide (Ce_2_O_3_), and titanium oxide (TiO_2_) [4]. All of the compounds are capable of precisely providing plants with necessary nutrients as well as ensuring favorable conditions during their growth [5]. Nanotechnology offers a potential path towards exceptional quality of food products. There are currently a number of ongoing scientific studies aimed at increasing the applicability of nanotechnologies in plant production and processing [6,7]. Modern fertilizers are a necessary element of efficient agriculture, but at the same time, they pose the risk of reducing soil fertility by upsetting its mineral balance [8]. Nanoparticles are added to such preparations to maximize productivity, reduce nutrient losses, and increase yields while also allowing a reduction in the overall necessary chemical treatment [9]. The use of silver or copper nanometals in traditional cultivations of oleaginous plants (e.g., rapeseed), counted among the most basic sources of nutrition worldwide, remains insufficiently explored in terms of both plant response and impact on the physicochemical properties of end products (oils). One should bear in mind that nanoparticles are small and have a relatively large surface area, which facilitates slower and extended release of nutrients to reduce wastage during plant growth [10]. As follows from the literature reports, some plant oils show antioxidative properties, reducing the production of free radicals under the influence of UV radiation and atmospheric pollutants. The free radicals present in the latter can penetrate the epithelium, altering its structure and attacking the DNA, aromatic amines, and polyunsaturated fatty acids, which exacerbates skin ageing and the risk of cancer. Moreover, vegetable oils are rich in valuable unsaturated omega-3, omega-6, and omega-9 fatty acids which help to protect the organism against skin dehydration. They contain compounds such as phytosterols, tocopherols, vitamins C and E, carotenoids, bioflavonoids, and polyphenols [11,12], as well as carbohydrates (squalene), chlorophylls, and other pigments showing high antioxidative activity [13]. The content of said compounds in oil depends on its quality, type of raw plant material, as well as weather conditions during cultivation and agrotechnical treatments employed during production [14,15]. Alongside overall lifestyle and general living conditions, diet is among the key factors influencing human health and wellbeing. It is therefore unsurprising that consumers increasingly choose food types with proven health benefits such as cold-pressed vegetable oils which are a rich source of the aforementioned antioxidants [16,17]. 

Given the observed lack of publications that would fully explore the antioxidative properties of oils in the content of nanoparticles and their use, in our study, we aimed to conduct analyses that would evaluate the impact of metal nanoparticles (silver and copper) on winter rapeseeds grown and harvested during a 2-year field experiment conducted in 2019 and 2020. The seeds obtained from experimental cultivations were tested in terms of their basic chemical parameters (fat and protein content) and cold-pressed to produce oil (similarly analyzed for raw fat and protein content). The obtained oils were then examined to determine their content of phytochemical compounds and their antioxidative potential, with a particular focus on the phosphorus (P) content, as well as the acid number (AN) and peroxide number (LOO) parameters. Further analyses determined the composition of fatty acids, content of sterols and squalene, tocopherols, total chlorophyll, carotenoids, and total polyphenols, as well as overall antioxidative activity (DPPH^•^). Subsequently, the oil samples underwent a qualitative analysis with the use of FTIR spectroscopy measurements as a noninvasive, fast, and very reliable method of identifying general differences between organic samples of this type.

## 2. Materials and Methods

### 2.1. Origin of the Material

The following research hypothesis was adopted: the application of silver and copper nanocolloids on winter rape plants at phenological development stages of plants (BBCH 12-16) and (61-66 BBCH) contributes to the improvement of phytochemical and antioxidative properties of cold-pressed oil. 

The experiments were conducted on unenclosed seeds of spring rape (*Brassica napus* L. var.arvensis f. annua) of the ‘Markus’ cultivar obtained from Hodowla Roślin Strzelce Sp. z o.o. Grupa IHAR.

The nanomaterial for laboratory and field experiments included two commercially available compounds sold in 1 dm^3^ bottles containing nanosilver and nanocopper colloids at the concentration of, respectively, ≥0.1% silver (ITP-1KAg PO) and ≥0.1% copper (ITP-1KCu PO), produced by ITP-SYSTEM Sp. z o.o. in Dąbrowa Górnicza, Poland. 

### 2.2. Cultivation of Rapeseeds

The field temperament was conducted at a farm in Czesławice, Lubelskie Voivodeship, Poland—51°18′23″ N 22°16′02″ E. 

In the two-year field experiment (2019–2020), unenclosed winter rapeseeds (*Brassica napus* L. var.arvensis f. annua) of the ‘Markus’ cultivar were used. The plants were sprayed with nanoparticles concentrated at 50 mL/L to prevent any potential harm to the natural environment. The seeds were sown in triplicate onto experimental plots of 30 m^2^. Two experimental variants were explored: in the first, the plants were sprayed with metal nanocolloids only during the BBCH 12-16 growth phase, whereas in the second, the growing plants were sprayed twice, during the BBCH 12-16 and BBCH 61-66 (florescence) stages. The control sample was sprayed with neither silver nor copper nanocolloids. Following plant emergence, the number of plants per m^2^ was regulated to 60. For mineral fertilization, a multi-component NPK fertilizer was applied prior to sowing, dosed at 60 kg P_2_O_5_, 90 kg/ha K_2_O and complemented with nitrogen (N) fertilization at 92 kg/ha. Herbicide protection was provided with Clomazone dosed at 96 g/ha. During vegetation, in the BBCH31 stage BCH31 phase, the plants were sprayed with a liquid fertilizer containing 150 g of boron (B) per liter, in the form of boron ethylamine dosed at 1 L/ha. 

In the last week of July, the rape was harvested in a single-stage system. The harvested seeds were winnowed and stored in jute sacks for two weeks, in laboratory conditions, at 20 °C, with daily stirring to equalize the moisture levels in the entire bulk of the material. Over the three-year period, the average yield from the control field was 1.5 t/ha. 

### 2.3. Scanning Electron Microscopy (SEM)

The SEM method was used to analyze the size and morphology of the preparations containing silver and copper nanoparticles. The test samples were rinsed twice with distilled water and ultrasonicated, then placed in round aluminum samples, slightly dried and placed in the chamber of the scanning electron microscope—Quanta 3D FEG (FEI).

Micrographs were taken with an ETD detector at accelerating voltage of 30 kV. The AgNPs and CuNPs diameter was measured with Nis-Elements Advanced Research software 4.50. Particle 104 distribution was derived from a histogram generated for 300 particles.

### 2.4. Preparation of Nanoparticle Solutions 

Preparations containing nanoparticles (silver nanoparticles and copper nanoparticles) and intended for spraying were prepared at the concentration of 50 mL/L (0.005%) by mixing a suitable amount of the preparation volumetrically in 1 L of distilled water. 

### 2.5. Measurement of the Mass of One Thousand Seeds (MTS)

The mass of one thousand seeds (MTS) was measured by randomly selecting and weighing seeds within 0.01 g, using an AS 110 R2 electronic scale by Radwag. The analysis was conducted in triplicate for each experimental variant.

### 2.6. Analysis of the Protein Content in the Seeds and Dry Material 

The protein content was calculated using Kjeldahl’s method, through mineralization the sample in concentrated sulfuric acid (VI) in the presence of catalysts, alkalization of the solution, distillation of NH3, and titration of ammonia bound with boric acid with sulfuric acid, in accordance with the PN-A-04018:1975/Az3:2002 standard [18]. The results were expressed in % and estimated as the mean from three measurements.

### 2.7. Analysis of the Fat Content in the Seeds and Dry Material 

A Soxtec 8000 device (ASN 310 applications) was used to conduct analyses of fat content in rapeseed samples, in accordance with the PN-EN ISO 659:2010 standard [19].

The method entails multiple, continuous extractions of fat from pulverized and pre-dried product using an organic solvent, then removing the solvent and weighing the fatty substance. The results are expressed as % and estimated as the mean from triplicate measurements.

### 2.8. Oil Extraction Process

Milk thistle and borage oil. The seeds were pressed using a Farmet DUO screw press (Czech Republic) with the capacity of 18–25 kg/h and engine power of 2.2 kW. Four-kilogram batches of seeds were pressed using a nozzle with a diameter of 10 mm. Before beginning the process, the press was heated up to 50 ± 1 °C. The temperature was measured with an Ama-digit thermometer. After pressing, the oils were set aside for 7 days to allow natural sediment deposition.

### 2.9. Analysis of Fatty Acid Composition

Fatty acids were analyzed by way of gas chromatography to determine the qualitative and quantitative composition of the mixture of fatty acid methyl esters prepared in accordance with PN-EN ISO 12966-2:2011 [20].

### 2.10. Determination of FFA, Phosphorus and Water

The content of FFA in the oil samples was determined in accordance with PN-EN ISO 660 [21]. The content of phosphorus was determined in accordance with PN-ISO 10540-1 [22]. The content of water was measured with the Karl Fischer method. 

### 2.11. Chlorophyll and Carotenoid Pigments Content

Chlorophyll and carotenoid content was also measured in the rapeseed oil samples. This was conducted in cyclohexane in accordance with a slightly modified protocol originally described by Chtourou et al. The absorbance of each sample was measured at 670 nm for chlorophyll and 470 nm for carotenoids using a spectrophotometer (UV-2600i, Schimadzu, Japan). Chlorophyll and carotenoid content is given in mg per g of oil [23].

### 2.12. Determination of Flavonoids and Polyphenols

The tested rapeseed oil samples weighing 2.5 g were dissolved in 5 mL of hexane and extracted sequentially with three portions of 90% aqueous methanol (3.5 mL). The hydrophilic layer, filtered through a 0.45 µm polytetrafluoroethylene (PTFE) syringe membrane filter (Merck Millipore, Poland), was collected in a round-bottom flask and dried in a rotary vacuum evaporator (SBS-RV-5000, Steinberg Systems, Germany) at 38 °C. The remaining dried material was dissolved in 1.5 mL of methanol and stored at 20 °C until the analysis. A spectrophotometric measurement of total phenols was performed as described by Siger et al. [24], while total flavonoids were determined with the method described by Choo et al. [25]. The measurements were conducted using a spectrophotometer (UV-2600i, Schimadzu, Japan).

### 2.13. Total Antioxidant Capacity DPPH

The anti-radical activity of the analyzed oil samples was determined with the 2,2-diphenyl-1-picrylhydrazyl (DPPH) test, in accordance with the method described and developed by Prescha et al. [26]. The determinations were performed using a spectrophotometer (UV-2600i, Schimadzu, Japan).

### 2.14. Determination of the Content of Phytosterols and Squalene

The determination of phytosterol and squalene content was performed in accordance with the method described by Shuklę et al. [27]. Separation was achieved using the technique of gas chromatography. The gas chromatographer (AgilentGC7890B) was coupled with a 7000D mass spectrometer (Agilent Technologies, Santa Clara, CA, USA). An Elite-17ms capillary column (PerkinElmer, Waltham, MA, USA, 30 m × 0.25 mm × 0.25 µm) was used. Helium with the flow intensity of 1.1 mL/min was used as support, and the separation was performed at temperatures from 120 °C (for 1 min) to 290 °C (for 5 min); the temperature was increased at the rate of 5 °C/min. The temperatures of the source and the transfer line were, respectively, 230 °C and 290 °C. Quantitative measurements were performed using a 6890 N gas chromatographer (Agilent Technologies, SantaClara, CA, USA) equipped with an FID detector and an Elite-17ms capillary column (PerkinElmer, Waltham, MA, USA, 30 m × 0.25 mm × 0.25 µm). As the internal reference for the quantitative analysis, 5α-cholestane was used, and the calculations were performed using Chemstation v. B.04.02 software. In the case of co-separated peaks, we used Amdis ver. 2.66 software (NIST, Gaithersburg, MD, USA) with a database of mass spectra measured for pure standard samples (.ims and .isl) to separate the mass spectra, and identify and quantify co-interspersed molecules. The determination of phytosterol and squalene content was performed in accordance with the method described by Shukla et al. [27], with slight modifications. Separation was achieved using the gas chromatography technique. The gas chromatograph (AgilentGC7890B) was coupled to a 7000D mass spectrometer (Agilent Technologies, Santa Clara, CA, USA). An Elite-17ms capillary column (PerkinElmer, Waltham, MA, USA, 30 m × 0.25 mm × 0.25 µm) was used in the determination. Helium with a flow rate of 1.1 mL/min was used as the carrier and the separation was carried out within the temperature range of 120 °C (for 1 min)–290 °C (for 5 min); the temperature was increased at a rate of 5 °C/min. The source and transfer line temperatures were 230 °C and 290 °C, respectively. Quantitative measurements were performed using a 6890 N gas chromatograph (Agilent Technologies, SantaClara, CA, USA) equipped with an FID detector and an Elite-17ms capillary column (PerkinElmer, Waltham, MA, USA, 30 m × 0.25 mm × 0.25 µm). As an internal reference for quantitative analysis, 5α-cholestane was used, and calculations were performed using Chemstation v. B.04.02 software. For co-separated peaks, we used Amdis ver. 2.66 software (NIST, Gaithersburg, MD, USA) with a database of mass spectra measured for pure reference samples (.ims and .isl) to separate mass spectra and identify and quantify co-occurring molecules.

### 2.15. Determination of the Content of Tocopherols

In accordance with the protocol described by Fromm et al. [28], the content of tocopherols was determined by saponifying the oil samples and subsequently separating them in an Acquity CSH 130 UPLC C18 column (1.7µm, 1.0 × 100 mm, Waters, Milford, USA) using an Acquity Waters UPLC PDA system (Waters, Milford, USA).

### 2.16. Acid Number and Peroxide Number Measurements

The evaluation of oil quality included determination of the acid number (AN) by titration in accordance with DIN EN ISO 660:2005 [29], and of the peroxide number (LOO), also by titration, in accordance with DIN EN ISO 3960:2005 [30].

### 2.17. Oxidative Stability Measurements

The oxidative stability of the tested rapeseed oil samples was measured using a Rancimat 670 apparatus (Metrohm AG, Herisau, Switzerland). Test samples of 2.5 g were weighed on an analytical balance (AS 220.R2 PLUS, Radwag, Poland), placed in reaction vessels and heated to 120 °C under a 20 L/h dry air stream. The volatile compounds released during oxidation were collected in a cell that contained distilled water, and the increasing water conductivity was measured on a continuous basis. The time required to reach the conductivity infiltration point was recorded as the induction period (IP) expressed in hours. All determinations were carried out in triplicate.

### 2.18. FTIR Measurements 

The measurements of infrared FTIR spectra (ATR-FTIR, Attenuated Total Reflectance–Fourier Transform Infrared Spectroscopy) with background correction (40 scans per sample) were performed using a dedicated spectrometer extension—QATR-S Single Reflection ATR ACCESSORY. The extension utilizes a diamond prism. Measurements of all the spectra for the analyzed oil samples were performed at room temperature T = 23 °C. The spectra were measured using an IRSpirit spectrometer from SHIMADZU, Japan. Before measurements for each consecutive sample, the crystal was cleaned using ultrapure solvents. All solvents were purchased from Sigma-Aldrich. Measurements were performed at the resolution of 4 cm^−1^ within the spectral range of 4000–300 cm^−1^. The spectra were Fourier transformed and subsequently averaged, then analyzed and prepared for publication in Grams/AI 8.0 software (Thermo Fisher Scientific, Waltham, MA, USA). All the measurements were performed in triplicate, at the Department of Biophysics, University of Life Sciences in Lublin, Poland. 

### 2.19. Statistical Analysis

In order to determine the significance of the impact that the respective factors had on the analyzed values, a two- and three-way variance analysis was performed. The significance of differences between the mean values was established with Turkey’s test. 

In order to establish dependencies between the respective parameters, Pearson’s correlation analysis was performed. The statistical analysis was conducted using Statistica 13 software from StatSoft. The adopted significance level was *p* ≤ 0.05. All the tests and analyses were conducted in 5 replications.

### 2.20. Multivariate Statistical Analysis

For multivariate analysis, Statistica 13 (TIBCO Software Inc. PaloAlto, CA, USA) and OriginPro (OriginLab Corporation, Northampton, MA, USA) were used. Before chemometric analysis, the obtained FTIR spectra were preprocessed using OriginPro and Grams/AI 8.0 software (Thermo Fisher Scientific, Waltham, MA, USA). Subsequently, preprocessing steps including Savitzky–Golay smoothing (5-point window, second order polynomial), multi-point baseline correction, Y offset correlation and mean center were used. After the data processing step, unsupervised methods such as Principal Component Analysis (PCA) and Hierarchical Clustering Analysis (HCA) were employed in the wavenumber region of 1850–500 cm^−1^. 

PCA is one of the most popular multivariate methods of reducing a large set of correlated variables to uncorrelated latent variables called principal components (PS). Each PC is a particular linear combination of the original quality characteristics and still explains all the variance in the matrix of the original variables [31]. However, the principal components are determined in such a way that the first PC explains the largest part of the observed variability, and each subsequent one is orthogonal to the same, and represents a smaller part of the variance. Principal component analysis is a multivariate technique that enables one to classify samples, evidence patterns, and explore general relationship between dependent variables. The PCA is an exploratory technique based on the following expression (1):

X = TP^T^ + E, (1)

where X is the data matrix to be analyzed, T is called score matrix, P is the loading matrix, and E is the residual. 

HCA is an exploratory method, the purpose of which is to classify objects into groups (clusters) calculated from distance matrix and the similarity between them. HCA is based on determining the smallest distances between items (such as spectroscopic spectra) and the measure of dissimilarity between sets of observations. Those objects with the highest degree of similarity will be clustering into the same group. Dissimilar items will be placed in another cluster. Tree diagram obtained from hierarchical clustering analysis is called a dendrogram. In HCA, Euclidean distance between the pairs of samples was used as a distance measure and complete linkage criteria were used as an agglomeration method. 

## 3. Results 

### 3.1. Scanning Electron Microscopy

**Figure 1:**
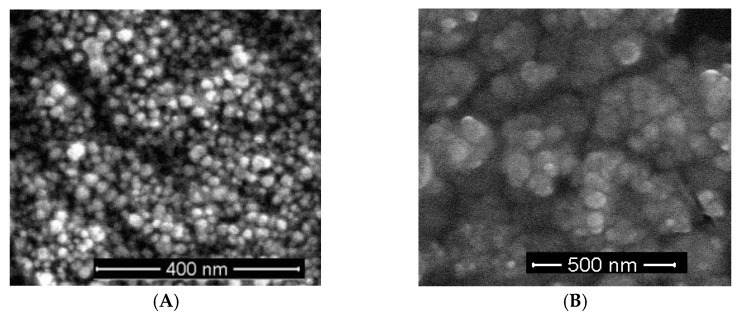
(**A**) SEM micrograph of AgNPs taken at ×100,000 magnification; (**B**) SEM micrograph of CuNPs taken at ×100,000 magnification.

The sizes of nanoparticles used in the study were estimated using scanning electron microscopy (Figure 1) and averaged at, respectively, 13.3 ± 3.6 and 38.3 ± 10.9 nm for AgNPs and CuNPs. Evaporation of the liquid in which nanoparticles were suspended prior to the SEM measurement caused natural precipitation of nanoparticle aggregates. Hence, in order to achieve a high degree of dispersion of the analyzed nanoparticles, the colloid samples were exposed to ultrasounds prior to measurement (as described in the methods section). In the case of silver nanoparticles, the images show a clearly granular structure without evident signs of agglomeration. However, despite using a similar methodology, complete deagglomeration of the copper nanoparticle sample was not achieved. The above observations suggest that nanoparticle sizes measured in the SEM experiment will be slightly overestimated due to the surface charges and flattening effects [32]. 

### 3.2. The Basic Chemical Composition of Seed Analysis 

In 2019, the mean fat content in the control sample was 44.07% (Table 1). After the (x1) and (x2) application of the silver nanoparticle treatment, the same decreased by 1.0% and 0.33%, respectively. In turn, the application of CuNPs resulted in a slight increase in the parameter, respectively, by 1.72% and 0.68% for the (x1) and (x2) application. 

The mean protein content in the control sample rapeseeds was 21.57%. The application of both nanoparticle treatments in both doses led to a decrease in this value. After the (x1) and (x2) application of AgNPs, the protein content decreased by 0.28% and 0.32%, respectively. In the case of CuNPs, the corresponding decrease was 4.36% and 4.73%, respectively. 

MTS analyses performed for the control sample yielded the result of 4.59 g. The mass of one thousand seeds was observed to decrease only after the (x1) application of AgNPs with the result 3.49% lower compared to the control sample. After the (x2) application of nanosilver, the seed mass increased by 11.55%. After the application of copper nanoparticles in that year, the MTS parameter also increased relative to the control sample, respectively, by 4.36% after single application, and 11.11% after double application of CuNPs. 

The mean fat content (42.66%) in the seeds harvested in 2020 increased in all experimental samples treated with nanocolloids compared to the control sample. The increase in the respective variants ranged from 0.39% (x2 AgNPs) to 2.11% (x1 CuNPs).

The mean content of protein in the control sample seeds was 21.96%. In the analyzed experimental variants, the value was decreased. The protein content decrease ranged from 1.21% after the single and double (x1 and x2) application of AnNPs to 5.61% after the single (x1) application of CuNPs. 

Determination of the MTS provides information about the quality of the material harvested in a given period. Based on the obtained results, it can be observed that the mass of one thousand seeds decreased in the samples subjected to the nanoparticle treatments. The observed seed mass decrease ranged from 4.56 g after (x1) application of AgNPs to 4.65 g after (x2) application of CuNPs. The greatest decrease in the MTS value was observed for the (x2) AgNPs variant, where it was 18.61% lower than in the control sample. 

### 3.3. The Physicochemical Parameters in Oil

The table below (Table 2) presents the basic parameters of the oil, specifically the acid, peroxide, and iodine value, phosphorus content, and oxidative stability. In the rapeseed oil obtained from plants harvested in 2019, the acid number (AN) was 0.60 mgKOH/g in the control sample. The application of AgNPs and CuNPs increased that value. Relative to the control, the AN increased by 22.78% and 36.11% for AgNPs and 67% and 80% for CuNPs, respectively, for the particular dosage regimens. 

In oil samples obtained in 2020, the acid number measured for the control was 0.66 mgKOH/g, i.e., 9.5% higher relative to the previous year. After the application of AgNPs and CuNPs, both in the (x1) and (x2) variant, the values were increased by 22.78%, 36.11%, and 67.78%, 80.0%, respectively, compared to the control sample. 

The control peroxide number parameter (LOO) was 1.29 meqO_2_/kg in 2019. Each (x1) and (x2) application of AgNPs and CuNPs resulted in increase in that value. For AgNPs, the LOO value was, respectively, 3.36% and 2.58% higher compared to the control. For CuNPs, the corresponding increase was recorded at 6.72 and 8.27%.

In 2020, the LOO of the control oil was 1.31 meqO_2_/kg, which was 1.77% higher compared to the previous year. The application of both types of nanoparticles during the plant growth period increased the value of this parameter measured in the resulting oil. The measured levels were, respectively, 3.36% and 2.58%, and 6.72% and 8.27% higher. 

In 2019, the iodine value (IV) measured in control rapeseed oil was 152.93 gI_2_/100 g. In experimental samples obtained after the application of metal nanoparticles, the corresponding levels were increased. The observed values were higher, as compared to the control sample, by, respectively, 0.85% and 1.48% for AgNPs, and 4.73% and 6.04% for CuNPs. In the following year (2020), the IV level in the control sample was 155.16 gI_2_/100 g, i.e., 1.43% higher compared to 2019. Overall, the application of metal nanoparticles increased the levels of this parameter in both experimental years in nearly all experimental variants, with the respective increase ranging from 1.18% for the (x2) AgNPs treatment in 2020 to 6.04% for the (x2) CuNPs treatment in 2019. The only exception was the (x2) CuNPs variant in 2020, for which an 8.49% decrease relative to the control was observed. 

The content of phosphorus in the 2019 control oil sample was 19.7 ppm. After the application of silver and copper nanometals, the value of the parameter increased in all experimental variants. The AgNPs treatment increased the phosphorus content by 97.5% (both at the x1 and x2 dose). The application of nanocopper also significantly increased the content of phosphorus in oil, respectively, by 23.35% (x1) and 74.62% (x2).

In the subsequent year of cultivation (2020), the parameter measured for the oil pressed from rapeseeds behaved entirely differently. The content of phosphorus in the control sample was 18.77 ppm, which was 24.50% lower compared to the previous year. Moreover, the application of nanometals in both experimental variants further decreased the same in the experimental oil samples. After spraying the plants with AgNPs, the respective values were 18.77% (x1) and 51.72% (x2) lower compared to the control. For CuNPs, the corresponding phosphorus levels decreased by, respectively, 3.1% (x1) and 17.24% (x2).

The oxidative stability index for the control sample was 5.94 h in 2019. After the application of metal nanoparticles, the same was increased in both experimental variants. The respective increase after the (x1) and (x2) treatments was 2.81% and 1.29% for AgNPs, and 4.94% and 8.31% for CuNPs.

In 2020, the overall oxidative stability of the analyzed oil decreased and was 5.66 h for the control sample, i.e., 0.39% shorter than in the preceding year. The application of the analyzed nanoparticles during the plant growth stage extended the period of oxidative stability. In the oil obtained from plants treated with AgNPs, the increase was 2.57% (x1) and 4.81% (x2). After the CuNPs treatment, the obtained values were, respectively, 9.56% and 12.07% higher than those of the control. 

### 3.4. Content of Fatty Acids

Table 3 presents the results in terms of the content of fatty acids in the analyzed oil samples. In both experimental years, 7 fatty acids were identified in the samples, namely palmitic acid C16:0, stearic acid C18:0, oleic acid C18:1n9c, linoleic acid C18:2n-6, linolenic acid C18:3, arachidic acid C20:0, and eicosenoic acid C20:1. In both experimental years, in most cases, the levels of respective acids in the oil samples obtained from plants treated with metal nanoparticles increased compared to the control. 

In the first year of cultivation (2019), the level of C16:0 decreased by 1.13% relative to the control in the (x1) AgNPs variant. In the case of C18:2, a noticeable decrease of 0.38% was recorded after the (x2) application of AgNPs. The content of C20:1 in rapeseed oil also decreased after the (x1) application of both AgNPs and CuNPs as well as (x2) CuNPs. The respective decrease was 0.55%, 0.55%, and 1.64% relative to the control. 

In the subsequent year (2020) of harvest, analyses of the oil samples in most cases revealed decreased levels of the respective fatty acids. Similar values were recorded in terms of the C18:3 content in all experimental variants, 1.71% and 1.71% for (x1) and (x2) AgNPs, respectively; and 3.33% and 3.36% for (x1) and (x2) CuNPs, respectively. The total content of Omega acids n3/n6/n9 in the first year of the experiment (2019) was higher in every variant of the experimental nanoparticle treatment compared to the control. The greatest increase (0.57%) was observed after the (x1) and (x2) application of CuNPs. However, in 2020, the results were quite the opposite, with Omega acid levels decreasing in all variants of the experiment. The greatest decrease relative to the control was recorded for the (x1) and (x2) application of CuNPs where the content was, respectively, 0.89% and 1.10% lower compared to that of the control.

### 3.5. The Total Content of Tocopherols in Oil

The total mean content of tocopherols in the 2019 control sample was 872.58 mg/kg (Table 4). After the (x1) and (x2) application of silver nanoparticles, the value increased by 1.3% and 0.5%, respectively. The CuNPs treatment also resulted in an increase in terms of this parameter, respectively, by 1.9% (x1) and 1.6% (x2) relative to the control. Tocopherol β was not detected in any of the analyzed oil samples. 

In 2020, the mean total content of tocopherols in the control oil sample was 878.69 mg/kg, i.e., 0.70% higher than in the preceding year. After the (x1) and (x2) application of silver nanoparticles, the same increased by 0.6% and 0.3%, respectively, relative to the control. The corresponding use of copper nanoparticles also statistically increased the content of total tocopherols, respectively, by 2.03% and 1.7% relative to the control. Similar to the previous year, tocopherols from the β group were not detected in the samples. 

In 2019, the free radical scavenging capacity of the studied oils, determined using the common DPPH^•^ antioxidant methods, was 1.80 mg Trolox/100 g in the control sample. The (x1) and (x2) use of AgNPs nanoparticles increased that capacity by 6.67% and 3.33%, respectively, relative to the control. The (x1) and (x2) treatment with CuNPs also increased the values of the parameter, respectively, by 21.11% and 11.67% compared to the control. 

In 2020, the free radical scavenging capacity measured for the control sample was 1.88 mg Trolox/100 g, i.e., 4.25% higher than in the preceding year. The (x1) application of both silver and copper nanoparticles increased the corresponding values of the parameter, but after (x2) spraying, the resulting values were decreased in both variants. In the former case (x1), the capacity increased by 2.66% for AgNPs and 12.43% for CuNPs; in the latter case (x2), the corresponding values were 4.15% and 4.33% lower relative to the control. 

### 3.6. The Content of Polyphenols and Flavonoids in the Analyzed Oils

Table 5 presents the content of polyphenols and flavonoids in the analyzed oils. In the first year of cultivation (2019), the polyphenol content in the control sample was 2.91 mgGAE/g of oil. Spraying the plants with the two experimental mixtures increased the value of this parameter. After the (x1) and (x2) application of AgNPs, the registered polyphenol content was 36.12% and 10.44%, respectively, higher than the control. In the case of CuNPs, the corresponding increase reached 78.56% and 69.04%, respectively. 

In 2020, the polyphenol content in the control sample was measured at 3.18 mgGAE/g, which was 8.5% higher compared to the preceding year. Also in this year, the silver and coppern nanocolloid treatment increased the parameter’s value. For AgNPs, it was 30.85% and 33.16% higher, respectively, and for CuNPs, the respective values were 33.16% and 55.82% higher relative to the control sample. 

In 2019, the content of flavonoids (Table 5) in the control sample was 21.67 mg/kg. In the oil obtained from plants sprayed with AgNPs (x1) and (x2), the same was higher by 5.55% and 2.66%, respectively, compared to the control. After the application of CuNPs, the value also increased, by 7.14% and 6.46%, respectively. In the subsequent year of the experiment (2020), the content of flavonoids in the control sample was 22.15 mg/kg, i.e., 2.18% higher compared to the preceding year. After the application of the tested mixtures containing metal nanoparticles, the flavonoid levels increased relative to the control, by 4.71% and 3.96%, respectively, for AgNPs and 9.00% and 7.97, respectively, for CuNPs. 

Table 5 presents the results regarding the content of chlorophylls and carotenoids in the oil samples. The control oil obtained from seed harvested in 2019 contained 1.31 mg/kg of chlorophyll. After the application of AgNPs, the value increased by 8.88 and 10.91%, respectively. The oil obtained from plants treated with CuNPs, the analyzed parameter increased considerably more than in the case of nanosilver, specifically by 21.83% and 29.95%, respectively. In the subsequent year of the experiment (2020), the oil pressed from the control seeds contained 1.24 mg/kg of chlorophyll, i.e., 5.91% more than in the previous year. After the (x1) and (x2) treatment with AgNPs, the content of chlorophyll in the resulting oil was 9.95% and 13.4% higher, respectively, relative to that of the control. In oil samples obtained from plants treated with CuNPs, the corresponding values were also increased—29.03% (x1) and 36.83% (x2) higher than those of the control. 

### 3.7. The Contents Sterols and Squalene in the Plant Oils

Table 6 presents the basic sterols present in the plant oils. The total content of respective sterols varied between the samples. In 2019, the mean total content thereof in the control sample was 558.37 mg/100 g of oil. In oils obtained from plants subjected to the experimental treatment, the highest content was observed for β-sitosterol, Δ^5^-Campestanol, Δ^5^-Avenasterol. The content of cholesterol was decreased. The values relative to the control sample differed depending on the treatment dosage. In the case of β-sitosterol, the content increased, respectively, by 7.87% (x1), 7.38% (x2) for AgNPs, and 9.47% and 9.85% for CuNPs. For Δ^5^-Campestanol, the value increased by 0.60% (x1) and 0.77% (x2) for AgNPs, and by 2.34 and 2.50% for CuNPs. Δ^5^-Avenasterol values increased by 0.33 and 0.57% (AgNPs) and 0.99% and 1.03% (CuNPs). In the case of cholesterol, the decrease relative to the control was, respectively, 0.74% (x1) and 1.19% (x2) for AgNPs, and 1.32 and 1.65% for CuNPs. In the subsequent year of the experiment (2020), the mean content of total sterols in the control sample was 560.84 mg/100 g of oil—0.44% higher compared to the preceding year. Again, the treatments increased the β-sitosterol, Δ^5^-Campestanol, and Δ^5^-Avenasterol content, while the content of cholesterol was decreased. 

The content of squalene in the 2019 control sample was 44.86 mg/100 g of oil. The application of nanoparticles increased said content in the experimental oil samples. Treatment with AgNPs increased the level of squalene by 13.31% and 14.07%, respectively. Oil from plants treated with CuNPs contained, respectively, 19.30% and 16.27% more oil than the control. In 2020, the experimental treatment also increased the levels of squalene in respective oil samples, specifically by 11.86% (x1) and 7.04% (x2) for AgNPs, and 23.07% and 22.66% for CuNPs.

Pearson’s analysis of linear correlations (Table 7 and Table 8) between the analyzed factors corroborated our expectations and revealed a close dependence between the content of tocopherols *δ*, *γ* and *α*, on the one hand, and DPPH (0.61; 0.79; 0.77), flavonoids (0.81; 0.95; 0.84), squalene (0.84; 0.91; 0.81), chlorophylls (0.83; 0.70; 0.91), carotenoids (0.85; 0.70; 0.94), LOO, AN, and oxidative stability on the other. The analysis performed with respect to the year of cultivation and oil production, content of fatty acids and antioxidative compounds in oil also revealed a high level of dependence (Table 8). 

### 3.8. FTIR Results

**Figure 2:**
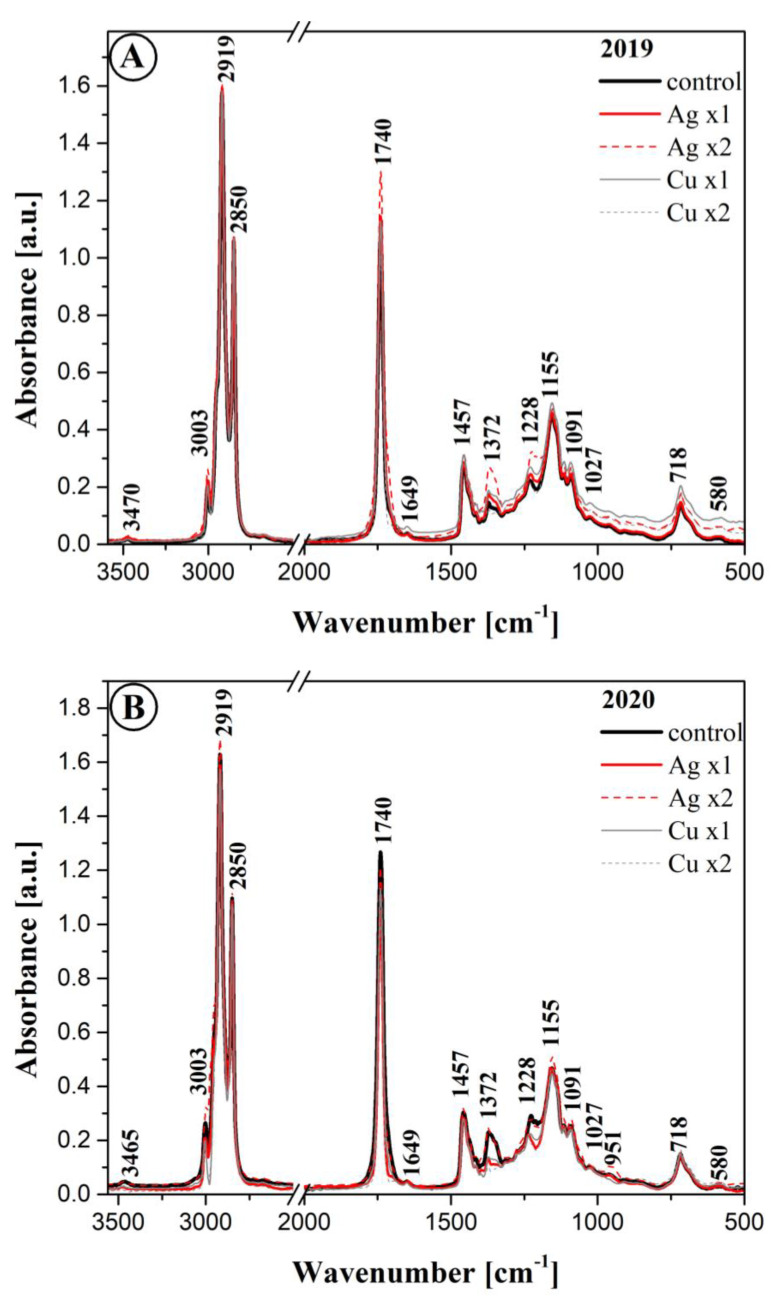
FTIR spectra for the analyzed oil samples obtained in 2019 (Panel **A**) and 2020 (Panel **B**), measured within the spectral range of 500 to 3600 cm^−1^. Spectra measured at room temperature (see Materials and Methods).

Figure 2 presents FTIR spectra recorded for selected samples of oil cold-pressed from rapeseeds harvested from plants sprayed with solutions containing silver and copper nanoparticles over the course of a two-year field experiment, as reflected in the already discussed results. To facilitate easier analysis and comparison of the samples, all the spectra were normalized to the same intensity with the maximum at ~1741 cm^−1^. In turn, Table 9 describes all the characteristic bands identified in the analyzed spectra, along with the identification of the corresponding functional group vibrations. The assignment of specific vibrations to the bands was performed on the basis of a detailed literature review [33,34,35,36,37,38,39,40]. As follows from the literature, most edible vegetable fats, including oleaginous materials, are substances that contain various fractions of triglyceride groups [40]. The literature also provides valuable data allowing identification of specific bands in the spectra recorded for oils of plant origin [38,39,40,41,42,43,44,45,46,47].

### 3.9. Multivariate Analysis

**Figure 3:**
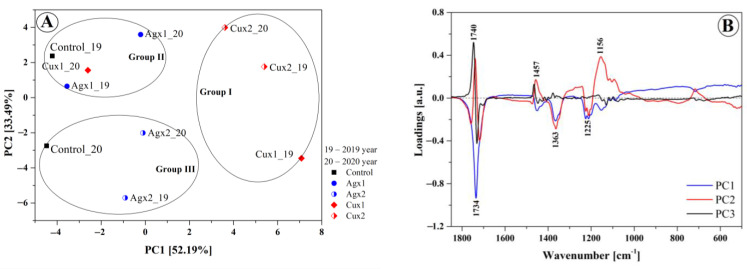

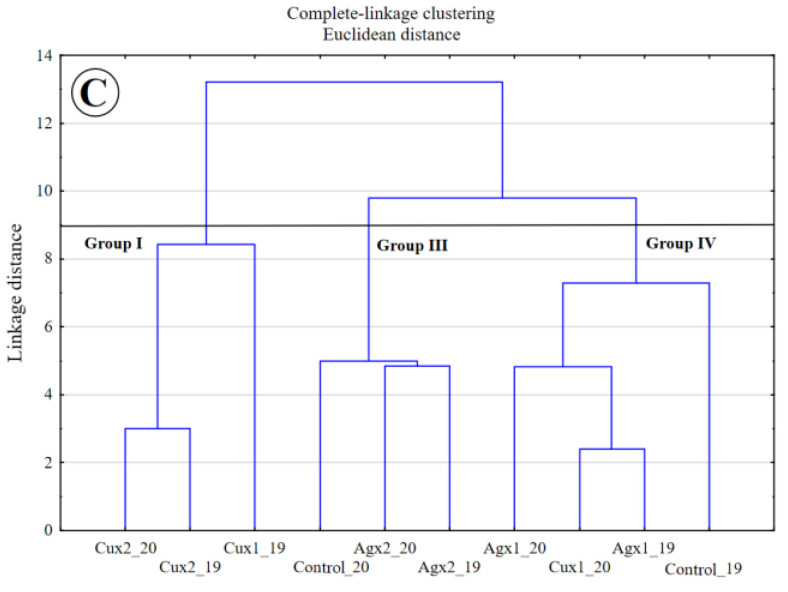
PCA and HCA analysis of the FTIR spectra of the analyzed oil samples. The PCA score plot (**A**) and loadings (**B**). A dendrogram tree (**C**) from the HCA. Euclidean distance between the pairs of samples was used as a distance measure and the complete linkage criteria was used as an agglomeration method. The designations 19 and 20 indicate samples obtained in 2019 or 2020, respectively.

For the purpose of this study, FTIR analysis was combined with chemometric approaches such as Principal Component Analysis (PCA) and Hierarchical Clustering Analysis (HCA) to obtain a general description of the samples’ distribution and possible grouping in homogeneous clusters. Regarding the evaluation of specific functional groups, interesting differences were identified in the fingerprint region, therefore an exploratory PCA and HCA data analysis was performed in the wavenumber range of 1850–500 cm^−1^. The resulting PCA scores and loading plots and HCA of the FTIR spectra can be seen in Figure 3. The eigenvalues and contributions of total variance obtained from principal component analyses of the FTIR spectra are presented in Table 10. The first two principal components explained over 85% of the total variance and underwent further study. Figure 3A presents a score plot in a two-dimensional projection for all the studied samples. The first two PCs clearly separate oils obtained from cultivations sprayed with solution containing Ag or Cu nanoparticles. The formation of three clusters can be observed: the first cluster, positively correlated with PC1, contained most of oil the samples with (x2) application of CuNPs obtained in 2019 and 2020 (Cux2_19, Cux2_20) and (x1) application of CuNPs obtained in 2019 (Cux1_19). The second and third clusters, both negatively corelated with PC1, mostly contained the samples with (x1) and (x2) application of AgNPs and control samples obtained in the two experimental years (Control_19, Control_20). Moreover, oil samples fertilized with nanocopper and sprayed either once (x1) or twice (x2) were grouped at opposite sides relative to PC2. The PCA loadings plot (Figure 3B) indicates that the maximum contribution to spectral differences was due to changes in the vibrations associated with carbonyl group C=O stretching from the fatty acid (1740–1705 cm^−1^) and C-O stretching modes (1156 cm^−1^) from esters. The arrangement of points on the score plot (Figure 3A) is related to the differences in the intensities for the aforementioned spectral regions.

A hierarchical cluster analysis was conducted on the same data set in the wavenumber range of 1850–500 cm^−1^, and the results are shown on a dendrogram in Figure 3C. Considering the cut of 9.0 dissimilarity units, three clusters are distinguished. As can be observed, the groupings for the HCA and PCA analyses are very comparable. The dissimilarity of respective clusters was defined by Euclidean distance and calculated with the complete-linkage method. The first group contained samples fertilized with nanocopper (Cux2_19, Cux2_20 and Cux1_19). The second and third clusters were composed of control samples and samples fertilized with nanosilver in 2019 and 2020. The hierarchical cluster analysis revealed that the grouping closely depended on the type of fertilization and amount of the nanocolloidal solution used (x1 or x2).

## 4. Discussion

Studies are conducted worldwide with a view to verifying the impact of nanoparticles on plant growth. The results published so far have reported contradictory effects in terms of plant toxicity, biomass accumulation, developmental stages, or accumulation of nutrients depending on the particular type of plant and nanoparticle in question [48]. 

The overall yield, mass of 1000 seeds, content of raw fat, total protein and glucosinolates are all characteristics that tend to differentiate seeds of different rape cultivars. The size of seed is an important factor in the oil production industry as oil from smaller seeds tends to contain more phosphorus and its nonhydratable forms, which are particularly difficult to eliminate. The mass of 1000 seeds (MTN) registered in the experiment was varied. Measured for the control sample, it was lower in the first year of the cultivation (4.59 g) and higher in the second (4.88 g). The obtained results were lower than those advertised by IHAR-Hodowla Roślin Strzelce Sp.z o.o. for this cultivar. In 2019, the MTN values in the experimental samples ranged from 4.43 after single application of AgNC to 5.12 g also for AgNC but with double application (x2); and in 2020, the valued ranged from 4.56 for (x1) AgNC to 4.88 g (the control). As we have not been able to identify papers discussing nanoparticle fertilization of growing rape plants, we could only relate those results to standard rape cultivations. The results reported by Murawa and Warmiński [49] pertained to different rape cultivars (“Star” and “Margo”). In their experiment, the authors used a variety of protective agents. According to their report, the mass of 1000 seeds did not differ between the respective years of the study but, depending on the combination of agents used, ranged from 4.05 g (after desiccation and pest control) to 4.53 g (after using Butisan + Ronilan).

Other crucial parameters influencing the quality of rapeseeds intended for the production of food oil include fat and protein content which depend, to varying degrees, on a combination of agrotechnical, climatic, and cultivar-related factors [50]. The content of those ingredients in seeds and seed yields is significantly influenced by weather conditions, particularly precipitation [51,52]. Dry mature rapeseeds contain primarily oil (45–50%) and protein (20–25%). Fat production industry is, quite understandably, most interested in the high oil content in the harvested seeds, but the protein fraction can also have certain uses, e.g., in the fodder industry [53]. The protein content depends primarily on the level of fertilization, cultivar, and weather. In our study, we observed significant discrepancies between the two years of the experiment as well as under the influence of the nanoparticle treatments. In 2019, the lowest fat content was registered in seeds after a single application of AgNP (43.63%), and the highest after a single application of CuNP (44.83%). In 2020, the fat content ranged from 42.66% in the control sample to 43.56% in the sample treated with (x1) CuNP. In a field experiment entailing the use of a fertilizer combination containing sulfur and nitrogen conducted by Barczak et al. [54], the average fat content in rapeseeds was comparable to our results, ranging between 35% and 56.7%.

Availability of protein is the primary factor influencing the growth and development of all organisms. As to its presence in rapeseeds, it has been empirically demonstrated that the quality of rape protein is comparable to protein found in milk or soya [55,56]. It is adequately capable of providing the amino acids necessary in human nutrition [57]. In both years of our experiment (2019 and 2020), we observed the highest content of protein in the seeds harvested from plants subjected to a single or double CuNP treatment, respectively, 20.63% and 20.55% in 2019, and 20.73% and 21.60% in 2020. Our results were comparable to those reported by other authors. In a study by Balalić et al. [58] conducted on a variety of rapeseed cultivars, the protein content also varied depending on the year of cultivation. It ranged from 22.15% in the first year to 18.78% in the second. In a study by Šidlauskas and Rife [59], the biannual average of protein content ranged from 19.97% to 21.54%. 

Rapeseed oil is one of the most commonly consumed vegetable oils, mainly due to its high content (approx. 90%) of 18-carbon unsaturated acids. Moreover, it is rich in many bioactive compounds whose presence in food and value is currently under intensive investigation. Many of the same are antioxidants. Oil is also a source of necessary unsaturated fatty acids from the n-6 and n-3 groups. The content of linolic and α-linoleic acid in rapeseed oil is usually approximately 20% and 10%, respectively [59]. Most authors agree that the correct n-6:n-3 ratio should be between 1:1 and 4:1 [60,61]. The ratio of n-6 to n-3 acids in diet should not exceed 4 [60,62].

It is important to facilitate the production of products with proven health benefits while at the same time promoting the development of sustainable agriculture. The results obtained for the control sample revealed that the most prevalent fatty acids included oleic acid C18:1 (16.29% in 2019, 16.51% in 2020), linoleic acid C18:2 (18.37%, 18.59%) and α- linolenic acid C18:3 (52.15%, 53.2%) both in the first and second year of cultivation. The application of nanoparticles during the plant growth stage contributed to increasing the content of said acids in almost every experimental variant analyzed. The only exception was observed in the case of C18:3 acid for which a decrease was recorded for every variant in 2020. The content of fatty acids in oil obtained from plants subjected to nanoparticle treatment was, in the case of Omega n-3 and n-6, higher than that reported by other authors. In rapeseed oil studied by Sagan et al. [63], the content of said acids was under 2.3%. Based on the literature data, it can be concluded that the content of n-6 acids in winter rapeseed oil tends to range between 5 and 5.5%, and of n-3 acids between 4.5 and 6% [64]. 

Rapeseed oils are also characterized by good oxidative stability, better than that of soybean or sunflower oil [65]. The oxidative stability of rapeseed oil can be further improved through supplementation with natural antioxidants present in spices [66]. Tocopherols contained in vegetable oil are natural phenolic antioxidants as well as the main source of vitamin E in human diet. Their content in oil can vary greatly, from 70 to 1900 mg/kg [67]. Tocopherols in the seeds of oleaginous plants come in four distinct forms: α-, β-, γ-, and δ-. Of those, α-tocopherol is the most active form of vitamin E [68,69]. The mean total tocopherol content in our oil control samples in both years of the experiment (2019, 2020) was higher than 872 mg/kg and increased depending on the nanoparticle treatment used during plant growth [70]. The values were also higher than those reported for sunflower (737.00 mg/kg) or rape (822.80 mg/kg) oil in a study conducted by Ergönül et al. [70]. Based on the current reports, the content of respective α-, β-, γ-, and δ-tocopherols depends on the type of the oil in question, cultivation techniques, as well as the oil pressing method employed [70]. In our study, β-tocopherol was not detected in any of the samples from either of the experimental years. In a study by Ergönül et al. [70], δ-tocopherol was not detected in sunflower, corn, or rapeseed oil. In a study by Wroniak et al. [71], the concentration of total polyphenols ranged between 51.73 and 70.30 mg/100 g. In other studies by Farhoosh et al. [72] and Gliszczyńska and Sikorska [68], the content of total tocopherol in ray soybean and rapeseed oil was, respectively, 983 mg/kg and 852 mg/kg. Swiglo and Skorska [68] reported the content of total tocopherol in refined corn and rapeseed oil was 815.80 mg/kg and 505.67 mg/kg, respectively.

Sterols play an active role in plants’ adaptation to biotic and abiotic stressors [73]. The sterols most commonly present in oil include β-sitosterol (75–90% of all sterols), Δ5-avenasterol (5–20%), campesterol (1–4%), and stigmasterol (0.5–2%), although the cited ratios may vary depending on the type of oil [74]. The compounds are present either as free sterols or in the form of sterol esters. After extraction from plant material, they are referred to as phytosterols [75]. Their presence in oil suggests the capacity to lower the levels of LDL cholesterol in blood by reducing cholesterol absorption, thus mitigating the risk of heart diseases [76,77]. It was also demonstrated that sterols of plant origin have anti-inflammatory and immunomodulatory properties, as well as the ability to reduce lipid accumulation on artery walls [78,79]. In our analyses, the mean sterol content in the control sample was similar in both years of the study, namely 558.37 (2019) and 560.84 (2020) mg/100 g of oil. After the application of metal nanoparticles, the levels increased in all experimental variants. Compared to results reported by other authors, the content of sterol in the analyzed samples was either lower or comparable. The total sterol content in rapeseed oil can be anywhere between 4500 and 11,300 mg/kg [75]. In a study by Fernandes et al. [76], the reported sterol content in raw oil was 820 mg/100 g of oil, and in refined oil it was 770 mg/100 g of oil. 

The content of squalene in raw vegetable oil is usually within the range of 1–3% of oil [80]. Squalene has beneficial properties, including antioxidant capacity, and has been claimed to reduce the risk of a number of cancers and lower the concentration of cholesterol in blood serum [81]; it has also been noted to show photoprotective, anticancer, and cardioprotective properties [82]. As observed by Lozano-Grande et al. [83], the levels of this component are highly variable and dependent on the agrotechnical conditions in particular cultivations. In our study, the mean control squalene content was 44.86 mg/100 g of oil in 2019 and 48.89 mg/100 g of oil in 2020, and increased depending on the nanoparticle treatment employed. The recorded values were comparable to or higher than those reported by other researchers. In a study by Nergiz [80], the content of squalene was shown to depend on the method of oil processing. In raw rapeseed oil, the level was 26.2 mg/100 g of oil and in refined oil it decreased to 24.2 mg/100 g of oil. 

Rapeseed oil is rich in phenolic compounds with natural antioxidant properties important to human health. Flavonoids and phenolic acids are among antioxidant defense systems protecting vegetable oils against oxidative damage. Numerous studies have explored the biological properties of polyphenol, which include anticancer, hypoglycemic, and anti-inflammatory activity. Apart from reducing the blood level of lipids, they also help to prevent cardiovascular diseases [84,85]. Such oils contain more polar phenols whose concentration ranges from 18 to 99 ppm of caffeic acid equivalents [86]. The combination of phenolic compounds and α-tocopherol is more effective in preventing lipid oxidation than the combination of phenolic compounds and vitamin C [87]. The results obtained in our study for cold pressed oil revealed that the content of polyphenols the first year of the experiment (2019) ranged from 2.91 in the control to 5.19 mg GAE/g of oil in the sample treated with x1 CuNP. In the subsequent year, the polyphenol content varied from 3.18 in the control to 4.95 mg GAE/g in the sample from the x2 CuNP variant. Compared to the results reported by other authors, the content of phenols in our rapeseed oil was higher. In a study by He et al. [88], who analyzed various oil pressing methods, the content of this oil component was significantly higher in hot-pressed oil as compared to other methods, reaching 0.064 mg/g^−1^, which suggests that polyphenols remain relatively stable in temperatures of up to 120 °C. As reported by Siger et al. [89] in a study on three selected cold pressed vegetable oils, the content of polyphenols in rapeseed oil was 1.28 mg/100 g, in soybean oil—1.44 mg/100 g, and in sunflower oil—1.19 mg/100 g. 

Carotenoids, similarly to chlorophylls, are found in thylakoids constituting lipid–protein structures of the inner chloroplast membrane [90]. The pigments participate in the transport of light energy to the photosynthetic reaction centers. As observed by Foyery et al. [89], chlorophyll is one of the major pigments facilitating the capture of light photons and transfer of the excitation energy to the photosystem’s reaction center, where electrons are emitted to be used in further stages of photosynthesis. Indeed, overly reduced levels of chlorophyll affect the entire process of photosynthesis and cause excessive electrons to bind with oxygen molecules. Farghaly and Nafady [90] suggest that techniques entailing the application of AgNC led to a significant stimulation of pigment content (Chl b and carotenoids) in tomatoes, while only an insignificant increase was recorded for Chl a. In a study by Pradhan et al. [91], plants subjected to a CuNC treatment reacted positively even at the dose of 1 mg·dm^−3^ by producing higher amounts of chlorophyll. This is an important result as it evidences the fact that CuNPs have a significant impact on photosynthesis, seeing as Chl a is a key element of the photosynthetic pathway [92]. In a study by Taran et al. [93], the application of silver and copper nanocolloids concentrated at 120 mg·dm^−3^ resulted in increased levels of chlorophyll a and b, as well as carotenoids. 

A growing body of publications describe studies conducted with the use of a range of spectroscopic techniques, including FTIR infrared spectroscopy, on various vegetable oils such as rapeseed oil. Typically, the main structural differences include the degree and form of saturation of the acetyl groups of which the particular products are composed, as well as the length of their hydrocarbon chains [39]. Upon analyzing the ATR/FTIR spectra registered for the oil samples obtained in 2019 and 2020 from plants treated with Cu and Ag nanoparticles, we can notice a number of changes in specific spectral ranges. The discrepancies observed in the experimental samples primarily reflect the aforementioned changes in oil composition, with a particular focus on the content of fatty acids. Due to the sheer amount of fatty acid molecules, vibrations originating from other oil components tend to be overshadowed.

As the first step in the analysis, we proceeded to generally characterize the spectra recorded for all the samples. Considerably more evident discrepancies were observed in the so-called spectral fingerprint region, particularly between 1400 and 1050 cm^−1^. As such, by handpicking the most relevant vibrations observed in the spectra, we first noticed the very intensive vibrations characteristic of the methylene group. Their maxima are typically located within the range of 1350–1175 cm^−1^ [38]. They are stretching vibrations associated with the C-H in the -CH_3_ methyl grouping. Additionally, deformation vibrations of the same group are observed at ~1155 cm^−1^ and with the maximum at ~1372 cm^−1^. 

In turn, the stretching vibration of the ester bond, i.e., ν(C-O), is composed of two asymmetric vibrations. In our case, they were related to the C-C(=O)-O and O-C-C groups [44]. The intensity of the former vibration was noticeably higher [41], which seems to be a typical feature in samples of this origin. The bands associated with these vibrations were found in the ~1300 region for the C-C(= O)-O grouping, and with the maximum at ~1000 cm^−1^. In the spectra registered for the oil samples, the vibrations at 1320 served more as an enhancement of the band at 1372 and 1091 cm^−1^. Next, the bands characteristic of saturated esters, i.e., the vibrations of the C-C(=O)-O group are often found within the range of 1240–1160 cm^−1^ [38,40]. In our samples, they were present at ~1228 cm^−1^. Vibrations originating from unsaturated esters are usually found at much lower wavenumbers [38]. 

The O-C-O band, normally associated with primary alcohols, is found in the range of 1090–1020 cm^−1^, in our case at ~1091 cm^−1^. As for secondary alcohols, the corresponding band has the maximum at ~1100 cm^−1^. In the samples analyzed in this study, the same was mostly an enhancement of the band with the maximum at ~1155 cm^−1^. The described types of esters are naturally found in the molecules of triglycerides present in the studied oils. The band with the maximum at ~1228 cm^−1^ is often associated exclusively with the so-called out-of-plane deformation vibrations of the methylene group [45]. Moving forward to the region of vibrations with the maximum at ~1457 cm^−1^ and ~1315 cm^−1^ (the already mentioned widening), we can describe vibrations of methyl groups in the aliphatic chains of the oils [38,45]. Above 1000 cm^−1^, at ~ 900 cm^−1^ all the samples included a band originating from the stretching vibrations of cis- substituted olefin groups [38]. This band could also be enhanced by vibrations of the vinyl group [40]. As follows from numerous publications [38,39,40,41,42,43,44,45,46,47,48,49], the fingerprint region as well as the high wavelength ranges occurred where the most evident effects in terms of spectral intensity could be identified and associated with different concentrations of certain fatty acids, most likely C16 and C18:0, or C18:1 and C18:2, as well as C20:0, i.e., the primary components of the samples in question.

Another very characteristic spectral region containing the bands with the maximum at ~1740 cm^−1^ was associated with the stretching vibrations of the carbonyl group [39], which prove very reliable in studies and evaluations of this type of food products, e.g., in the assessment of ageing effects. The band is enhanced at ~ 1705 cm^−1^, which is a very characteristic feature that is often overlooked in band descriptions. It also corresponds to the vibrations of a carbonyl group, however, in this case primarily the one found in acid groups [38,39,40,41] which may evidence the presence of hydrogen bonds between the components of the given product in the form of -C=O**^…^**H-O-.

The next band with the maximum at 1649 cm^−1^ corresponded to vibrations associated with the stretching vibrations of the -C=C- group (in the cis- transformation) [38,42]. Another characteristic region contained deformation vibrations originating from the -C-H groups in -CH_2_ and -CH_3_ groupings (so-called scissor vibrations) with the maximum at ~1457 cm^−1^, already mentioned above. One should also note the vibrations in the region below 1000 cm^−1^. These corresponded to the out-of-plane deformation vibrations originating from cis- conformed -HC=CH- groups, as well as wagging vibrations of said groups, i.e., δ(-(CH_2_)*n-* and -HC=CH- cis—[38,40,42]. We observed certain subtle differences in terms of band intensity within the spectra, although this pertained primarily to the 2019 samples. Changes in band intensity in this type of samples can evidence slight differences in terms of bonds between individual structural units of fatty acids, or changes from cis- to trans- conformation in fatty acid chains. Interestingly, such changes were observed mainly in the 2019 samples, but were almost or completely non-detectable in the 2020 samples. 

At higher wavenumbers, samples of this type also tend to exhibit very characteristic vibrations, particularly the stretching vibrations of trans- -C-H groups with the maximum at ~3065 cm^−1^ belonging to the vibrations of the triglyceride fraction [38,46,47]; however, in the present case, the same were not particularly visible. Stretching vibrations of cis- C-H groups are characteristically linked with the bands with the maximum at ~3003 cm^−1^. The slight changes in band intensity observed ~3003 cm^−1^ and confirmed the differences in fatty acid content between the respective oil samples, especially those from plants sprayed with Ag or Cu nanoparticles. However, in this range the changes were not as clear. Bands with the maximum at ~2950, 2919 cm^−1^ originate for the stretching vibrations of the –C-H groups in -CH_3_ and -CH_2_ groupings. They belong to the aliphatic groups in triglycerides [36,38,47]. 

It is also noteworthy at this point that the observed spectral changes correlated very well primarily with the previously discussed changes in the fatty acid profile.

The content of other components discussed in this paper, such as chlorophylls, flavonoids, or polyphenols, is considerably less reflected by observable changes to infrared spectra. At best, they tend to be observable in enhancements of vibrations originating from particular fatty acids; however, as such, they would be rather difficult to meaningfully identify. 

## 5. Conclusions

The preliminary research hypothesis adopted in this study was corroborated. 

As follows from our experiments, in most cases, the use of the nanoparticle treatments had a positive impact on the phytochemical content of the analyzed components. The most noticeable negative effect may be associated with the size of the harvested seeds (MTS). In both years of the experiment, a reduction in terms of the mass of 1000 seeds was observed, regardless of the experimental variant. In 2019, MTS for the variant subjected to (x1) AgNPs was 3.49% lower compared to the control. In the following year, the MTS measured in all experimental variants was lower than in the control, with the decrease ranging from 4.60% (x2) CuNPS to 18.61% for (x2) AgNPs. 

The analysis of ATR-FTIR spectra measured for all the cold-pressed oils obtained from plants sprayed with solutions containing AgNPs or CuNPs revealed clearly visible changes in terms of intensity, most notably at 1740, 1372, 1228, and approximately 1090 cm^−1^. The changes reflected differences in the content of fatty acids in the particular oil samples and, albeit to a lesser extent, differences in the content of protein and other components of the oil. FTIR spectroscopy combined with principal component analysis (PCA) and hierarchical clustering analysis (HCA) successfully differentiated the rapeseed oil based on the type of fertilization and amount of nanocolloidal solutions used. 

## Figures and Tables

**Table 1:** Basic chemical composition of seeds on content fat, protein, and mass of one thousand seeds.

Year	Parameter	Unit	Control	AgNPs		CuNPs	
				x1	x2	x1	x2
2019	Fat	%	44.07 ± 0.15 ^def^	43.63 ± 0.15 ^bcde^	43.93 ± 0.15 ^cde^	44.83 ± 0.21 ^f^	44.37 ± 0.31 ^ef^
	Protein		21.57 ± 0.08 ^c^	21.51 ± 0.04 ^c^	21.5 ± 0.06 ^c^	20.63 ± 0.31 ^b^	20.55 ± 0.10 ^b^
	MTS	g	4.59 ± 0.23 ^a^	4.43 ± 0.23 ^a^	5.12 ± 0.25 ^abc^	4.79 ± 0.25 ^a^	5.10 ± 0.15 ^abc^
2020	Fat	%	42.66 ± 0.57 ^a^	43.10 ± 0.30 ^abc^	42.83 ± 0.43 ^ab^	43.56 ± 0.31 ^bcde^	43.46 + 0.35 ^abcd^
	Protein		21.96 ± 0.21 ^d^	21.70 ± 0.26 ^cd^	21.7 ± 0.26 ^cd^	20.73 ± 0.47 ^b^	21.60 ± 0.26 ^cd^
	MTS	g	4.88 ± 0.13 ^ab^	4.56 ± 0.28 ^a^	4.62 ± 0.45 ^a^	4.62 ± 0.34 ^a^	4.65 ± 0.44 ^a^

The results are mean values ± SD; values in same column designated with different letters indicate significant discrepancies (*p* ≤ 0.05).

**Table 2:** Primary physicochemical parameters in industrial oils.

Year		Acid Number (AN)	Peroxide Number (LOO)	Iodine Number (IV)	Phosphorus (P)	Stability Index
		mg KOH/g	meq O_2_/kg	g I_2_/100 g	ppm	h
2019	Control	0.60 ± 0.02 ^aa^	1.29 ± 0.02 ^aa^	152.93 ± 0.22 ^aa^	19.7 ± 0.10 ^aa^	5.94 ± 0.01 ^aa^
	Ag x1	0.73 ± 0.01 ^aa^	1.33 ± 0.01 ^aa^	154.23 ± 0.05 ^aa^	38.9 ± 0.33 ^ba^	6.10 ± 0.04 ^bb^
	Ag x2	0.81 ± 0.02 ^aa^	1.32 ± 0.01 ^aa^	155.2 ± 0.26 ^aa^	38.9 ± 0.32 ^ba^	6.01 ± 0.05 ^bb^
	Cu x1	1.01 ± 0.05 ^ba^	1.37 ± 0.01 ^aa^	160.16 ± 0.20 ^aa^	24.3 ± 0.42 ^aa^	6.23 ± 0.05 ^bb^
	Cu x2	1.08 ± 0.03 ^ba^	1.39 ± 0.01 ^aa^	162.16 ± 0.29 ^ba^	34.40 ± 0.51 ^ba^	6.43 ± 0.05 ^bb^
2020	Control	0.66 ± 0.02 ^aa^	1.31 ± 0.02 ^ab^	155.16 ± 0.29 ^aa^	26.1 ± 0.21 ^ab^	5.96 ± 0.01 ^aa^
	Ag x1	0.75 ± 0.01 ^ba^	1.35 ± 0.01 ^aa^	156.16 ± 0.25 ^aa^	21.2 ± 0.13 ^ab^	6.11 ± 0.05 ^ab^
	Ag x2	0.80 ± 0.01 ^ba^	1.37 ± 0.01 ^aa^	157.00 ± 0.50 ^aa^	12.6 ± 0.91 ^cb^	6.25 ± 0.01 ^ac^
	Cu x1	1.14 ± 0.02 ^ca^	1.41 ± 0.01 ^ba^	161.16 ± 0.29 ^ba^	25.3 ± 0.90 ^aa^	6.53 ± 0.05 ^bc^
	Cu x2	1.176 ± 0.01 ^ca^	1.41 ± 0.01 ^ba^	142.00 ± 0.21 ^cb^	30.6 ± 0.36 ^ba^	6.68 ± 0.02 ^bc^

The results are mean values ± SD; values in same column designated with different letters indicate significant discrepancies (*p* ≤ 0.05).

**Table 3:** Content of fatty acids in the analyzed oil samples (%).

Year		C16:0	C18:0	C18:1	C18:2	C18:3	C20:0	C20:1	∑n-6/n-3/n-9
2019	Control	6.06 ± 0.04 ^aa^	2.56 ± 0.03 ^ac^	16.29 ± 0.02 ^aa^	18.37 ± 0.05 ^aa^	52.15 ± 0.06 ^aa^	1.79 ± 0.02 ^aa^	1.81 ± 0.02 ^aa^	89.83
	Ag x1	6.15 ± 0.04 ^ba^	2.60 ± 0.02 ^ac^	16.32 ± 0.03 ^aa^	18.47 ± 0.02 ^aa^	52.89 ± 0.05 ^ba^	1.82 ± 0.02 ^ba^	1.84 ± 0.02 ^aa^	90.25
	Ag x2	6.18 ± 0.03 ^ba^	2.58 ± 0.02 ^ac^	16.32 ± 0.02 ^aa^	18.40 ± 0.01 ^aa^	52.69 ± 0.03 ^ba^	1.80 ± 0.01 ^ba^	1.79 ± 0.02 ^ba^	89.99
	Cu x1	6.41 ± 0.02 ^ba^	2.70 ± 0.02 ^bc^	16.56 ± 0.0 ^ba^	18.70 ± 0.04 ^aa^	52.38 ± 0.04 ^ba^	1.94 ± 0.02 ^ba^	1.82 ± 0.01 ^ba^	90.34
	Cu x2	6.27 ± 0.02 ^ba^	2.67 ± 0.02 ^bc^	16.44 ± 0.03 ^ba^	18.66 ± 0.01 ^aa^	53.11 ± 0.05 ^ba^	1.91 ± 0.01 ^ba^	1.80 ± 0.01 ^ba^	90.88
2020	Control	6.18 ± 0.03 ^ab^	2.53 ± 0.03 ^aa^	16.51 ± 0.05 ^ab^	18.59 ± 0.04 ^ab^	53.20 ± 0.03 ^ab^	1.89 ± 0.04 ^ab^	1.73 ± 0.04 ^ab^	90.83
	Ag x1	6.22 ± 0.02 ^aa^	2.63 ± 0.02 ^aa^	16.69 ± 0.0 ^a^	18.70 ± 0.02 ^aa^	52.29 ± 0.02 ^aa^	1.95 ± 0.02 ^bb^	1.78 ± 0.02 ^aa^	90.31
	Ag x2	6.18 ± 0.02 ^aa^	2.60 ± 0.02 ^aa^	16.70 ± 0.01 ^bb^	18.69 ± 0.02 ^aa^	52.29 ± 0.01 ^aa^	1.92 ± 0.01 ^aa^	1.75 ± 0.01 ^aa^	90.28
	Cu x1	6.50 ± 0.03 ^ba^	2.80 ± 0.03 ^ba^	16.79 ± 0.0 ^bb^	19.00 ± 0.03 ^aa^	51.43 ± 0.02 ^bc^	2.00 ± 0.02 ^ba^	1.84 ± 0.01 ^ba^	90.02
	Cu x2	6.41 ± 0.02 ^b^	2.74 ± 0.03 ^ba^	16.79 ± 0.01 ^bb^	18.89 ± 0.01 ^aa^	51.41 ± 0.01 ^bc^	1.95 ± 0.01 ^ba^	1.81 ± 0.01 ^ba^	89.83

The results are mean values ± SD; values in same column designated with different letters indicate significant discrepancies (*p* ≤ 0.05).

**Table 4:** Phytochemical compounds with antioxidant effects in oil.

Year	Tocopherols						DPPH
		Α	β	γ	δ	Total	
		mg/kg					mg Trolox/100 g
2019	Control	26.73 ± 0.25 ^ac^	-	831.13 ± 0.89 ^ac^	14.72 ± 0.03 ^ac^	872.58 ^ac^	1.80 ± 0.02 ^ac^
	Ag x1	27.97 ± 0.15 ^bc^	-	841.10 ± 0.40 ^bc^	14.82 ± 0.03 ^bd^	883.89 ^bc^	1.92 ± 0.02 ^bb^
	Ag x2	28.43 ± 0.06 ^be^	-	834.23 ± 0.65 ^ab^	14.89 ± 0.02 ^bd^	877.55 ^bd^	1.86 ± 0.02 ^ab^
	Cu x1	29.67 ± 0.15 ^bc^	-	844.73 ± 0.60 ^bc^	15.22 ± 0.03 ^bd^	889.62 ^bc^	2.18 ± 0.02 ^bc^
	Cu x2	29.85 ± 0.05 ^bc^	-	841.43 ± 0.61 ^bc^	15.55 ± 0.61 ^bd^	886.83 ^bc^	2.01 ± 0.03 ^bc^
2020	Control	27.13 ± 0.12 ^ab^	-	836.70 ± 0.46 ^aa^	14.86 ± 0.02 ^bc^	878.69 ^aa^	1.88 ± 0.02 ^aa^
	Ag x1	28.33 ± 0.03 ^bd^	-	840.97 ± 0.61 ^bd^	14.94 ± 0.01 ^bc^	884.24 ^bc^	1.93 ± 0.02 ^ab^
	Ag x2	28.52 ± 0.08 ^bd^	-	838.33 ± 0.47 ^ab^	15.08 ± 0.08 ^bc^	881.93 ^bc^	1.85 ± 0.02 ^ab^
	Cu x1	29.78 ± 0.10 ^bc^	-	851.03 ± 0.12 ^bd^	15.79 ± 0.02 ^bc^	893.68 ^bd^	2.08 ± 0.02 ^bd^
	Cu x2	29.87 ± 0.12 ^bc^	-	847.93 ± 0.59 ^bc^	15.88 ± 0.03 ^bc^	893.68 ^bd^	1.99 ± 0.01 ^bc^

The results are mean values ± SD; values in same column designated with different letters indicate significant discrepancies (*p* ≤ 0.05).

**Table 5:** Content of total polyphenols and flavonoids in the oils.

Year		Polyphenols	Flavonoids	Carotenoids	Chlorophylls
		mgGAE/g of Oil	mg/kg		
2019	Control	2.91 ± 0.02 ^aa^	21.67 ± 0.03 ^aa^	151.56 ± 0.71 ^aa^	1.31 ± 0.14 ^aa^
	Ag x1	3.95 ± 0.02 ^bb^	22.87 ± 0.03 ^bc^	155.50 ± 0.60 ^ab^	1.43 ± 0.02 ^ad^
	Ag x2	3.21 ± 0.04 ^bb^	22.25 ± 0.06 ^bc^	157.03 ± 0.25 ^bc^	1.45 ± 0.01 ^ac^
	Cu x1	5.19 ± 0.01 ^cd^	23.22 ± 0.04 ^cd^	160.86 ± 0.60 ^bd^	1.61 ± 0.01 ^bd^
	Cu x2	4.91 ± 0.04 ^db^	23.07 ± 0.03 ^cd^	165.13 ± 0.40 ^cd^	1.71 ± 0.02 ^cd^
2020	Control	3.17 ± 0.05 ^aa^	22.15 ± 0.05 ^ab^	153.78 ± 0.10 ^aa^	1.24 ± 0.02 ^ab^
	Ag x1	4.15 ± 0.05 ^bb^	23.21 ± 0.04 ^bd^	155.36 ± 0.13 ^ab^	1.36 ± 0.01 ^bc^
	Ag x2	4.23 ± 0.04 ^bc^	23.03 ± 0.03 ^bd^	156.68 ± 0.13 ^bc^	1.41 ± 0.01 ^bc^
	Cu x1	4.23 ± 0.03 ^be^	24.15 ± 0.04 ^cd^	161.33 ± 0.12 ^cd^	1.60 ± 0.01 ^cd^
	Cu x2	4.95 ± 0.05 ^bb^	23.92 ± 0.03 ^bd^	162.17 ± 0.08 ^cd^	1.69 ± 0.02 ^cd^

The results are mean values ± SD; values in same column designated with different letters indicate significant discrepancies (*p* ≤ 0.05).

**Table 6:** Content of sterols and squalene (mg·100 g^−1^ oil) in oil.

		Unit	2019					2020				
			Control	Ag x1	Ag x2	Cu x1	Cu x2	Control	Ag x1	Ag x2	Cu x1	Cu x2
Sterols	Δ^5^-campestanol	mg/100 g of oil	119.1 ± 0.14 ^aa^	119.81 ± 0.04 ^aa^	120.02 ± 0.06 ^aa^	121.89 ± 0.05 ^ba^	122.08 ± 0.08 ^ba^	119.46 ± 0.06 ^aa^	120.23 ± 0.11 ^aa^	120.51 ± 0.04 ^aa^	122.84 ± 0.13 ^aa^	123.24 ± 0.10 ^aa^
	campestanol		2.48 ± 0.03 ^aa^	3.28 ± 0.08 ^ab^	3.33 ± 0.03 ^aa^	3.41 ± 0.01 ^ba^	3.46 ± 0.02 ^ba^	2.6 ± 0.03 ^ab^	3.08 ± 0.03 ^ab^	3.21 ± 0.06 ^aa^	3.38 ± 0.15 ^ba^	3.45 ± 0.03 ^ba^
	β-sitosterol		315.48 ± 0.23 ^aa^	340.3 ± 0.16 ^ba^	338.76 ± 0.19 ^ba^	345.37 ± 0.08 ^ba^	346.54 ± 0.15 ^ca^	317.39 ± 0.15 ^ab^	341.56 ± 0.20 ^ba^	342.29 ± 0.15 ^ba^	347.11 ± 0.03 ^ca^	347.32 ± 0.03 ^ca^
	Δ^5^-avenasterol		55.11 ± 0.13 ^aa^	55.29 ± 0.03 ^aa^	55.42 ± 0.03 ^ba^	55.65 ± 0.08 ^ba^	55.68 ± 0.08 ^ba^	55.17 ± 0.05 ^aa^	55.48 ± 0.02 ^aa^	55.54 ± 0.01 ^aa^	55.89 ± 0.04 ^ba^	56.02 ± 0.04 ^bb^
	Δ^7^-stigmasterol		4.25± 0.05 ^aa^	4.33 ± 0.02 ^aa^	4.35 ± 0.01 ^aa^	4.42 ± 0.02 ^ba^	4.49 ± 0.02 ^ba^	4.26 ± 0.02 ^aa^	4.32 ± 0.01 ^aa^	4.36 ± 0.01 ^aa^	4.41 ± 0.04 ^ba^	4.46 ± 0.02 ^ba^
	cholesterol		33.56 ± 0.15 ^aa^	33.31 ± 0.03 ^aa^	33.16 ± 0.03 ^aa^	33.12 ± 0.03 ^aa^	33.01 ± 0.04 ^aa^	33.53 ± 0.04 ^aa^	33.49 ± 0.04 ^aa^	33.44 ± 0.04 ^aa^	32.97 ± 0.03 ^aa^	32.97 ± 0.01 ^aa^
	brassicasterol		28.38 ± 0.10 ^aa^	28.48 ± 0.03 ^aa^	28.69 ± 0.03 ^aa^	28.88 ± 0.04 ^aa^	28.97 ± 0.04 ^aa^	28.42 ± 0.02 ^aa^	28.49 ± 0.01 ^aa^	28.59 ± 0.03 ^aa^	28.67 ± 0.04 ^aa^	28.77 ± 0.03 ^aa^
	Total		558.37 ^aa^	584.81 ^ba^	583.75 ^ba^	592.76 ^ca^	594.24 ^ca^	560.84 ^aa^	586.66 ^ba^	587.96 ^ba^	595.29 ^ba^	596.24 ^ca^
squalene			44.86 ± 0.10 ^aa^	50.83 ± 0.31 ^ba^	51.17 ± 0.06 ^ca^	53.52 ± 0.05 ^da^	52.16 ± 0.05 ^ca^	48.89 ± 0.06 ^ab^	54.69 ± 0.03 ^bb^	52.33 ± 0.04 ^cb^	60.17 ± 0.05 ^db^	59.97 ± 0.04 ^db^

The results are mean values ± SD; values in same column designated with different letters indicate significant discrepancies (*p* ≤ 0.05).

**Table 7:** Results of Pearson’s liner correlation analysis with the significance threshold of *p* < 0.05.

Variables		DPPH	Flavonoids	Squalene	Chlorophylls	Phosphorus	Carotenoids	LOO	Oxidative Stability	AN
Tocopherols	α	0.776	0.846	0.815	0.916	0.220	0.946	0.910	0.863	0.952
	γ	0.791	0.958	0.911	0.700	0.043	0.709	0.862	0.841	0.842
	δ	0.618	0.847	0.847	0.835	0.078	0.857	0.883	0.965	0.952
Sterols		0.746	0.879	0.865	0.890	0.088	0.900	0.911	0.911	0.951
Polyphenols		0.853	0.913	0.810	0.812	−0.009	0.850	0.917	0.917	0.851

**Table 8:** Results of Pearson’s linear correlation analysis with the significance threshold of *p* <0.05.

	Variables	Tocopherols			Sterols	Polyphenols	Flavonoids	Squalene
		α	γ	δ				
	Year	0.786520	0.765176	0.816355	0.812864	0.772242	0.900074	0.912379
Fatty acids	C12:0	-	-	-	-	-	-	-
	C14:0	−0.471624	−0.446309	−0.307216	−0.368645	−0.420913	−0.481883	−0.496723
	C16:0	0.838304	0.906012	0.854864	0.908317	0.863860	0.855622	0.870484
	C16:1	-	-	-	-	-	-	-
	C18:0	0.848680	0.905633	0.871043	0.915687	0.891798	0.899430	0.870323
	C18:1	0.555212	0.726311	0.673462	0.657135	0.653637	0.801539	0.814341
	C18:2	0.735722	0.902980	0.854050	0.841343	0.826066	0.914740	0.899759
	C18:3	−0.394675	−0.551126	−0.585301	−0.575555	−0.451103	−0.611782	−0.668665
	C20:0	0.661390	0.804062	0.704169	0.723701	0.789544	0.835769	0.798867
	C20:1	0.274459	0.330049	0.257200	0.315330	0.346893	0.304878	0.251529

**Table 9:** Locations of FTIR absorption bands’ maxima registered within the spectral range of 500–3600 cm^−1^ and assignment of particular vibrations to the respective samples from 2019 and 2020—corresponding to data in Figure 2.

FTIR	Type and Origin of Vibrations
Positioning of Band [cm^−1^]	
3003	ν_m_(=C-H, cis-)
2919	ν_as, vst_(-C-H_vst_, -CH_2_) and ν_s, vst_(-C-H, -CH_2_) (aliphatic groups in triglycerides)
2850	
1740/1705	ν_vst_(-C=O) in esters / ν_vw_(-C=O) in acids
1649	ν_vw_(-C=C-, cis-)
1457	δ_vw_(-C-H) in CH_2_ and CH_3_ groups, deformation (scissor)/ ν_vw_(-C-H, cis-) deformation (wagging)
1372	ν_w, m, vw_(-C-H, -CH_3_) and deformation
1315	δ_m_(-C-H, -CH_3_)
1270	ν_m_(-C-O) or δ_m_(-CH_2_-)
1228	
1155	ν_st_(-C-O) or δ_st_(-CH_2_-)
1137	
1091	ν_m,vw_(-C-O)
1027	δ_w_(-HC=CH-, trans-) out-of-plane deformation
718	
580	

ν—stretching vibrations, δ—deformation vibrations, s—symmetric, as—asymmetric, st—strong, w—weak.

**Table 10:** Eigenvalues, percentage of variance, and cumulative percentage in the data used for the PCA calculations obtained from FTIR spectra of the to the analyzed oil samples.

Principal Component Number	Eigenvalue	Percentage of Variance (%)	Cumulative (%)
1	16.70399	52.19471	52.1947
2	10.71657	33.48589	85.6806
3	3.26747	10.20981	95.8904
4	1.00970	3.15501	99.0454
5	0.24075	0.75225	99.7977

## Data Availability

Not applicable.

## Acronyms

(x1)	single nanoparticle spray
(x2)	double nanoparticle spray
AgNP	silver nanoparticles
BBCH	phenological development stages of plants
CuNP	copper nanoparticles
MTS	mass of one thousand seeds
